# Additional challenges in reaching hepatitis C elimination goals in Germany due to the COVID-19 pandemic - descriptive analysis of drug prescription data from January 2018 to June 2021

**DOI:** 10.3389/fpubh.2023.1149694

**Published:** 2023-05-30

**Authors:** Emily D. Meyer, Sandra Dudareva, Christian Kollan, Stefan Mauss, Heiner Wedemeyer, Daniel Schmidt, Ruth Zimmermann

**Affiliations:** ^1^Department of Infectious Disease Epidemiology, Postgraduate Training for Applied Epidemiology (PAE), Robert Koch Institute, Berlin, Germany; ^2^European Programme for Intervention Epidemiology Training (EPIET), European Centre for Disease Prevention and Control (ECDC), Stockholm, Sweden; ^3^Department of Infectious Disease Epidemiology, Unit of HIV/AIDS, STI and Blood-borne Infections, Robert Koch Institute, Berlin, Germany; ^4^Center for HIV and Hepatogastroenterology, Düsseldorf, Germany; ^5^Department of Gastroenterology, Hepatology and Endocrinology, Hannover Medical School, Hanover, Germany

**Keywords:** hepatitis C, COVID-19, delivery of health care, Agenda 2030, antiviral agents - therapeutic use

## Abstract

Effectively treating hepatitis C viral (HCV) infections prevents sequelae and onward transmission. In Germany, HCV drug prescriptions have declined since 2015. During the COVID-19 pandemic, lockdowns impacted the access to HCV care services and HCV treatment. We assessed if the COVID-19 pandemic further decreased treatment prescriptions in Germany. We built log-linear models with monthly HCV drug prescription data from pharmacies from January 2018 - February 2020 (pre-pandemic) to calculate expected prescriptions for March 2020-June 2021 and different pandemic phases. We calculated monthly prescription trends per pandemic phase using log-linear models. Further, we scanned all data for breakpoints. We stratified all data by geographic region and clinical settings. The number of DAA prescriptions in 2020 (*n* = 16,496, −21%) fell below those of 2019 (*n* = 20,864) and 2018 (*n* = 24,947), continuing the declining trend from previous years. The drop in prescriptions was stronger from 2019 to 2020 (−21%) than from 2018 to 2020 (−16%). Observed prescriptions met predictions from March 2020 to June 2021, but not during the first COVID-19 wave (March 2020–May 2020). Prescriptions increased during summer 2020 (June 2020-September 2020) and fell below the pre-pandemic numbers during the following pandemic waves (October 2020 – February 2021 and March 2021 – June 2021). Breakpoints during the first wave indicate that prescriptions plummeted overall, in all clinical settings and in four of six geographic regions. Both, outpatient clinics and private practices prescribed overall as predicted. However, outpatient hospital clinics prescribed 17–39% less than predicted during the first pandemic wave. HCV treatment prescriptions declined but stayed within the lower realms of predicted counts. The strongest decline during the first pandemic wave indicates a temporary HCV treatment gap. Later, prescriptions matched predictions despite of pronounced decreases during the second and third waves. In future pandemics, clinics and private practices need to adapt more rapidly to maintain a continuous access to care. In addition, political strategies should focus more on continuously providing essential medical care during periods of restricted access due to infectious disease outbreaks. The observed decrease in HCV treatment may challenge reaching the HCV elimination goals in Germany by 2030.

## 1. Introduction

Hepatitis C virus (HCV) infections are a main cause of chronic viral hepatitis, liver cirrhosis and hepatocellular carcinoma (1–3). In the European Union and the European Economic Area, ~35,000 deaths were attributable to HCV infections in 2015 (4). In Germany, it was estimated, that 9,528 deaths were attributable to a hepatitis B or C infection in 2015 (4). Germany is considered a low-prevalence country for HCV due to a low prevalence in the general population (5). However, the prevalence is disproportionately higher among risk groups: the prevalence ranges between 9.6% for men who have sex with men (6) and 68.0% among people who inject drugs (5). As HCV infections account for a high burden of disease and deaths world-wide, the WHO adopted a global strategy to eliminate hepatitis B and C as a public health threat by 2030 as part of Agenda 2030 (7). To reach elimination, 90% of people living with an HCV infection should be diagnosed, and 80% of the diagnosed should be cured by 2030 (8). Germany adopted the elimination goal in the strategy BIS2030 (9).

The introduction of direct acting antivirals (DAAs) in 2014 lay the foundation for reducing the morbidity and mortality of HCV infections, e.g., by reducing liver decompensation events among hospitalized patients diagnosed with hepatitis C (10). Several DAA substances have been registered and approved in Germany, and since 2017 single-tablet pan-genotypic combination treatments are available (11). Standard treatment duration with these regimens ranges between 8 and 12 weeks. The aim of the treatment is viral clearance, defined as sustained virologic response 12 weeks after the end of the treatment, which is achieved in ~95% of treated patients (12).

HCV clinical care and treatment in Germany is mostly provided by specialist physicians, from either specialist practices for infectious diseases or gastroenterology or from specialized outpatient departments of tertiary care hospitals. From 2014 to 2020 the treatment indication for patients with statutory health insurance only included those with chronic HCV infections in Germany. As of November 2020 all viremic patients without clinical signs of acute HCV infection, are eligible for treatment with DAAs - irrespective of a proven chronic stage of the disease (12). The count of monthly DAA prescriptions increased between January 2014 and March 2015, but has decreased continuously, together with the number of treated patients, between March 2015 and December 2020 (13, 14). In recent years, treatment of HCV infection has become easier with the expanded use of pan-genotypic DAAs with a standardized treatment length. While high treatment costs were an initial barrier to prescribing treatment (13), average treatment regimen costs decreased over time from 91,000€ (2014) to 35,000€ (2018) and 30,700€ (2020) (14). For the combination of Elbasvir + Grazoprevir, the 12-week treatment costs less, ~26,000€ (15).

Globally, a ten-fold increase of HCV treated patients has been observed between 2015 and 2020, leading to a reduction in deaths attributable to HCV (16). However, HCV care deteriorated globally during the COVID-19 pandemic: non-pharmaceutical control interventions, e.g., lockdowns, physical distancing requirements and movement restrictions, but also disrupted supply chains and health services potentially slowed down or reversed the progress from previous years (16, 17). In Germany, the COVID-19 pandemic was divided into different waves (Supplementary material 1). According to Hüppe et al. (18), the first pandemic wave negatively influenced patient care: About 60% of all liver patient consultations in Germany between March and May 2020 were impeded because of reduced service availability and cancellations of consultations by patients (18). It was estimated that the diagnosis of liver decompensation and liver cancer was delayed by 22 and 9.4%, respectively (18).

We assessed the number of DAA prescriptions and treated HCV patients in Germany between January 2018 and June 2021, including whether DAA prescriptions between March 2020 and June 2021 were impacted by the COVID-19 pandemic. Based on this assessment, we discuss if Germany can reach the HCV elimination goals by 2030.

## 2. Materials and methods

### 2.1. Data

Insight Health^TM^ provided monthly prescription data for DAA to treat HCV-infected patients (19)The data are based on prescriptions redeemed by patients with statutory health insurance [88% of Germany's population (19)] between June 2018 to June 2021. The database comprises data from ~95% of German pharmacies. The DAAs comprised the following substances and combinations: DAA combinations effective against all genotypes (=pan-genotypic drugs) (Glecaprevir+Pibrentasvir, Velpatasvir + Sofosbuvir and Voxilaprevir + Velpatasvir + Sofosbuvir), DAA combinations effective against genotypes 1 and 4 (Elbasvir + Grazoprevir, Ledipasvir + Sofosbuvir) and others (Daclatasvir, Sofosbuvir, Dasabuvir, Ombitasvir + Paritaprevir + Ritonavir). Prescribed Ribavirin as potential part of some regimens was ignored, since it is only used in combination with DAAs in German treatment guidelines. All DAA are approved for treating adults; Glecaprevir + Pibrentasvir, Ledipasvir + Sofosbuvir are approved for treating children aged 12 years or older., We grouped the place of prescription in geographical regions: North (Lower Saxony, Bremen, Hamburg, Schleswig-Holstein, Mecklenburg-Vorpommern), South (Bavaria), Southwest (Baden-Württemberg, Saarland, Rhineland Palatinate, Hesse), East (Thuringia, Saxony, Saxony-Anhalt, Brandenburg, Berlin) and West (North Rhine, Westphalia-Lippe). Clinical settings were aggregated in outpatient clinics (for prescriptions that were not assigned to a specific independent physician) and private practices (for prescriptions from all specialized independent physicians).

We divided the observation period into pre-pandemic and pandemic periods based on a retrospective classification of the COVID-19 pandemic in Germany (20): Pre-pandemic period (January 2018–February 2020), first wave (March 2020–May 2020), summer 2020 (June 2020–September 2020), second wave (October 2020–February 2021) and third wave (March 2021–June 2021).

### 2.2. Data analysis

To calculate the number of treated HCV patients, the DAA prescription data were analyzed according to treatment regimen (12, 21). Based on the average treatment duration of different regimen, the number of patients treated with DAA and with statutory health insurance per year was estimated with weighted calculations of standardized treatment durations: with a standard regimen of 12 weeks for all DAA except for Glecaprevir + Pibrentasvir. For Glecaprevir + Pibrenasvir it was assumed that ~90% of the patients received DAA for 8 weeks and 10% for 12 weeks, similar to the 8.4 weeks published as the average treatment duration for Glecaprevir + Pibrenasvir by the German Hepatitis C Registry (22).

Based on pre-pandemic monthly data (January 2018–February 2020), we built a log-linear model with months as singly explanatory variable, with one unit representing 1 month, and predictions as the outcome to calculate the 80% prediction interval (23) for monthly DAA prescription counts. We considered this prediction interval to be more sensitive in detecting unexpected changes than a 95% prediction interval. We calculated the prediction intervals for the entire study period (March 2020–June 2021, start of first to end of third pandemic wave) and separately for each pandemic period. Observed prescription counts within the prediction interval were assigned a difference of 0 (0%). If observed prescription counts were below or above the prediction interval, we calculated the difference as counts and percent to the lower and upper 80% prediction interval bound.

To calculate monthly trends (changes in drug prescription in % per month) and their 90% confidence intervals, we built log-linear models based on data of the corresponding period. We chose a 90% confidence interval to increase the sensitivity in detecting changes from previous trends (24); as some pandemic waves lasted few months only, changes from trends might have been missed when using larger confidence intervals.

We scanned the log-linear models of the DAA prescription data from January 2018 to June 2021, independent from the pandemic periods, for structural breakpoints (25): a maximal number of ten structural breakpoints were assessed to detect all potentially relevant breakpoints. No other specific settings were used. The identified breakpoints were tested for significance with a Chow-Test and only those reaching a significance level of α ≤ 0.05 are presented.

We used R.4.0.5. (26) with the packages tidyverse, zoo, broom, MASS, janitor and strucchange (25) for analyzing the data.

### 2.3. Ethical statement

DAA prescription data are not individual data but monthly aggregated from pharmacies' billing data. No ethical clearance is needed for the analysis.

## 3. Results

### 3.1. Overview of DAA prescriptions in Germany from January 2018 to June 2021

During the study period, the total monthly DAA prescriptions halved from 2,293 in January 2018 to 1,108 in June 2021. Monthly DAA prescriptions ranged between 2,404 (March 2018) and 1,070 (January 2021). The decline in yearly prescriptions was more pronounced from 2019 to 2020 than from 2018 to 2019 (Table 1). During the first pandemic year, the number of DAA prescriptions decreased by 23% from 20,309 (March 2019–February 2020) to 15,699 (March 2020-February 2021). The decrease in DAA prescriptions from the first half of 2020 to the first half of 2021 was lower than previous yearly declines, except for the Western and Southern regions (Table 1). The estimated number of patients treated per year decreased from ~9,900 (2018) over ~8,100 (2019, −18%) to ~6,500 (2020, −20%).

**Table 1:** Yearly count of hepatitis C drug prescriptions in Germany from January 2018 to June 2021.

		**2018**	**2019**		**2020**		**2021 (Jan–Jun)**	
		**Prescriptions n**	**Prescriptions n**	**Difference to previous year n (%)**	**Prescriptions n**	**Difference to previous year n (%)**	**Prescriptions n**	**Difference to previous Jan–Jun n (%)**
Total prescriptions in Germany		24,947	20,864	−4,083 (−16%)	16,496	−4,368 (−21%)	7,356	−1,029 (−12%)
Provider	Private Practice	20,373	17,014	−3,359 (−16%)	13,715	−3,299 (−19%)	6,120	−945 (−13%)
	Outpatient clinics	4,574	3,850	−724 (−16%)	2,781	−1,069 (−28%)	1,236	−84 (−6%)
Region	North^1^	4,456	3,875	−581 (−13%)	3,024	−851 (−22%)	1,449	−65 (−4%)
	East^2^	2,903	2,413	−490 (−17%)	1,967	−446 (−18%)	971	−45 (−4%)
	West^3^	5,891	5,168	−723 (−12%)	4,347	−821 (−16%)	1,863	−353 (−16%)
	South^4^	4,036	3,337	−699 (−17%)	2,435	−902 (−27%)	942	−320 (−25%)
	Southwest^5^	7,629	6,058	−1,571 (−21%)	4,716	−1,342 (−22%)	2,131	−239 (−10%)

^1^Lower Saxony, Bremen, Hamburg, Schleswig-Holstein, Mecklenburg-Vorpommern. ^2^Thuringia, Saxony, Saxony-Anhalt, Brandenburg, Berlin. ^3^North Rhine, Westphalia-Lippe. ^4^Bavaria. ^5^Baden-Württemberg, Saarland, Rhineland Palatinate, Hesse.

**Figure 1:**
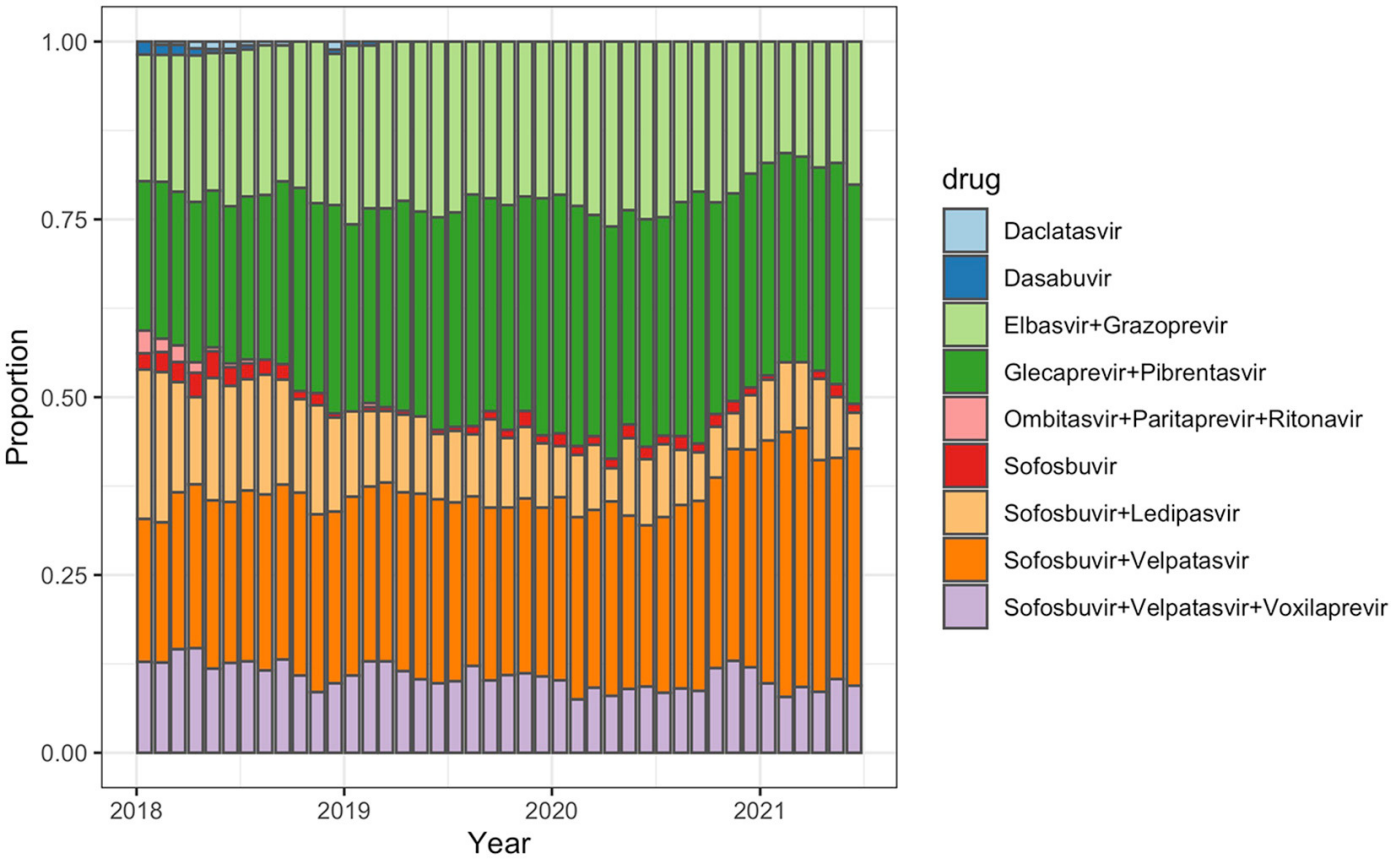
Monthly proportion of selected prescribed hepatitis C treatment regimens among all prescribed regimens in Germany between January 2018 and June 2021.

Prescriptions of pan-genotypic DAA combinations increased: from 73% of all prescribed DAA in January 2018 to 84% in June 2021 (Figure 1, Supplementary material 3). DAA that are effective against genotypes 1 and 4 played a minor role and their use decreased over time from 27% (January 2018) to 16% (June 2021). Other DAA (Daclatasvir, Dasabuvir, Paritaprevir + Ombitasvir + Ritonavir) were no longer prescribed after March 2019.

Between January 2018 and February 2020, the total monthly prescriptions declined by 2%. During the first wave, the total monthly prescriptions declined further (−19%); during summer 2020, a temporary increase was observed (+2%); followed by decreases during the second (−5%) and third (−10%) pandemic wave (Table 2).

**Table 2:** Mean monthly changes in the number of hepatitis C antiviral treatment prescriptions (in percentage to the respective previous month) in Germany between January 2018 and June 2021, stratified by pandemic phases.

		**Pre-pandemic^6^ % (90% CI^11^)**	**First wave^7^ % (90% CI)**	**Summer 2020^8^ % (90% CI)**	**Second wave^9^ % (90% CI)**	**Third wave^10^ % (90% CI)**
**Total prescriptions in Germany**						
		−2% (−2 to −1%)	−19% (−32 to −3%)	+2% (−7 to +12%)	−5% (−11 to +2%)	−10% (−15 to −4%)
Clinical setting	Practice	−2% (−2 to −1%)	−17% (−19 to −15%)	+2% (−4 to +9%)	−5% (−10 to +2%)	−9% (−16 to −2%)
	Outpatient clinics	−2% (−2 to −1%)	−27% (−84 to + 341%)	+1% (−20 to +28%)	−7% (−15 to +2%)	−11% (−16 to −5%)
Region	North^1^	−2% (−2 to −1%)	−18% (−50 to + 35%)	±0% (−20 to −25%)	−9% (−20 to +4%)	−17% (−32 to +3%)
	East^2^	−2% (−2 to −1%)	−16% (−23 to +3%)	±0% (−16 to + 20%)	−1% (−9 to +8%)	−9% (−29 to + 18%)
	West^3^	−1% (−1 to −1%)	−20% (−17 to +292%)	+3% (±0 to +7%)	−7% (−17 to +4%)	−10% (−15 to −5%)
	South^4^	−2% (−2 to −1%)	−18% (−50 to + 35%)	±0% (−20 to −25%)	−9% (−20 to +4%)	−17% (−32 to +3%)
	Southwest^5^	−2% (−2 to – 1%)	−19% (−38 to +5%)	+4% (−11 to + 22%)	−5% (−16 to + 8%)	−6% (−17 to +6%)

^1^Lower Saxony, Bremen, Hamburg, Schleswig-Holstein, Mecklenburg-Vorpommern. ^2^Thuringia, Saxony, Saxony-Anhalt, Brandenburg, Berlin. ^3^North Rhine, Westphalia-Lippe, ^4^Bavaria, ^5^Baden-Württemberg, Saarland, Rhineland Palatinate, Hesse, ^6^January 2018 to February 2020. ^7^March 2020 to May 2020, ^8^June 2020 to September 2020, ^9^October 2020 to February 2021, ^10^March 2021 to June 2021; ^11^Confidence interval.

### 3.2. DAA prescription trends during the pre-pandemic period

During the pre-pandemic period (January 2018–February 2020), the total monthly prescriptions declined by 2% (Table 2). The same trend was observed when stratifying for clinical settings and regions, except for the Western region (1%). In the South and East, prescriptions increased abruptly as of September 2018 with breakpoints of trends (*p* = 0.04 and *p* = 0.049, respectively (**Figure 3**).

### 3.3. Predicted and observed DAA prescriptions and their trends during the COVID-19 pandemic

Between the start of the first and the end of the third pandemic wave, the drug prescriptions in total stayed within the lower realms of predictions. Similarly, in most regions the observed prescriptions matched predictions (Table 3). In the Southern region, less DAA were prescribed than predicted. When stratifying for the clinical settings, the observed prescriptions matched predicted numbers (Table 3).

**Table 3:** Differences between observed and predicted Hepatitis C treatment prescriptions in Germany between March 2020 and June 2021 and stratified by pandemic phase with 80% prediction interval, based on the pre-pandemic prescription data.

		**March 2020 – June 2021**	**First pandemic wave^6^**	**Summer 2020^7^**	**Second pandemic wave^8^**	**Third pandemic wave^9^**
Total prescriptions in Germany	Observed	20,750	4,065	5,214	6,426	5,045
	Predicted 80% PI^10^	19,540-23,867	4,067 - 4,908	5,121 – 6,217	5,942-7,282	4,409-5,461
	Difference n (%)	**±0**	–**2 to** –**843 (-0.1 to -17%)**	**±0**	**±0**	**±0**
Practice	Observed	17,281	3,494	4,281	5,317	4,189
	Predicted 80% PI	16,121-19,486	3,350-4,002	4,222 – 5,073	4,905-5,947	3,644-4,464
	Difference n (%)	**±0**	**±0**	**±0**	**±0**	**±0**
Outpatient clinics	Observed	3,469	571	933	1,109	856
	Predicted 80% PI	3,281 – 4,572	690-941	864-1,191	995-1,394	733-1,045
	Difference n (%)	**±0**	–**119 to** –**370 (**–**17% to** –**39%)**	**±0**	**±0**	**±0**
North^1^	Observed	3,919	737	950	1,271	961
	Predicted 80% PI	3,201 – 4,434	676-918	844 – 1,158	969 – 1,350	711 – 1,008
	Difference n (%)	**±0**	**±0**	**±0**	**±0**	**±0**
East^2^	Observed	2,582	505	635	789	653
	Predicted 80% PI	2,043 – 2,842	431 – 588	539 – 742	619 – 866	454 - 647
	Difference n (%)	**±0**	**±0**	**±0**	**±0**	**+6 to** **+199** **(+1 to** **+44%)**
West^3^	Observed	5,372	1,052	1,386	1,616	1,318
	Predicted 80% PI	5,290 – 6,629	1,064 – 1,315	1,366 – 1,699	1,625 – 2,042	1,235-1,572
	Difference n (%)	**±0**	–**13 to** –**263 (-1 to -20%)**	**±0**	–**9 to** –**426 (-1 to -21%)**	**±0**
South^4^	Observed	2,913	613	786	861	653
	Predicted 80% PI	2,938-3,832	618-793	774-1,001	891-1,167	656 - 871
	Difference n (%)	–**25 to−919** **(**–**1 to−24%)**	–**5 to** –**180 (**–**1 to**–**-23%)**	**±0**	–**30 to** –**306 (**–**3 to** –**26%)**	–**10 to** –**209** **(**–**0.5 to** –**25%)**
Southwest^5^	Observed	5,958	1,155	1,454	1,889	1,460
	Predicted 80% PI	5,350 – 7,065	1,130-1,467	1,411-1,848	1,620-2,149	1,188-1,600
	Difference n (%)	**±0**	**±0**	**±0**	**±0**	**±0**

^1^Lower Saxony. Bremen. Hamburg, Schleswig-Holstein, Mecklenburg-Vorpommern. ^2^Thuringia. Saxony, Saxony-Anhalt, Brandenburg, Berlin. ^3^North Rhine. Westphalia-Lippe. ^4^Bavaria. ^5^Baden-Württemberg, Saarland, Rhineland Palatinate, Hesse. ^6^March-May 2020. ^7^June 2020-September 2020. ^8^October 2020-February 2021. ^9^March 2020-June 2021. ^10^80% prediction interval. The observed treatment prescriptions were expected to fall with a probability of 80% within the 80% prediction interval (PI), acknowledging that prescriptions vary slightly from month to month even without major events, e.g. a pandemic. If the number of observed treatment prescriptions fell within the 80% PI, a difference of ±0 was assigned. If the number of observed treatment prescriptions was outside of the 80% PI, the difference to both interval borders was calculated as a number and percentage.

**Figure 2:**
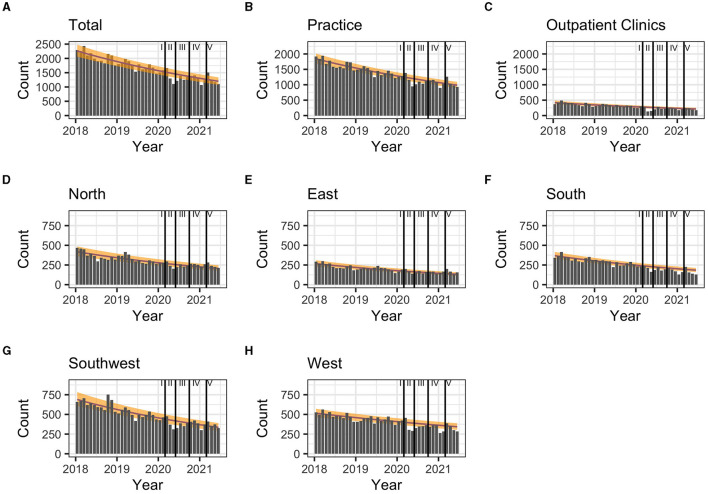
**(A–H)** Comparison of predicted (yellow band) and observed (gray bars) hepatitis C treatment prescriptions in Germany between March 2020 and January 2021 and by pandemic phase. 80% prediction interval based on pre-pandemic data (January 2018-February 2020). Regions: South: Bavaria; Southwest: Baden-Württemberg, Saarland, Rhineland Palatinate, Hesse; West: North Rhine, Westphalia-Lippe; North: Lower Saxony, Bremen, Hamburg, Schleswig-Holstein, Mecklenburg-Vorpommern; East: Thuringia, Saxony, Saxony-Anhalt, Brandenburg, Berlin. Pandemic phases: I: Pre-pandemic (January 2018 to February 2020), II: First pandemic wave (March 2020 to May 2020), II: Summer 2020 (June 2020 to September 2020), IV: Second wave (October 2020 to February 2021), V: Third wave (March 2021 to June 2021).

**Figure 3:**
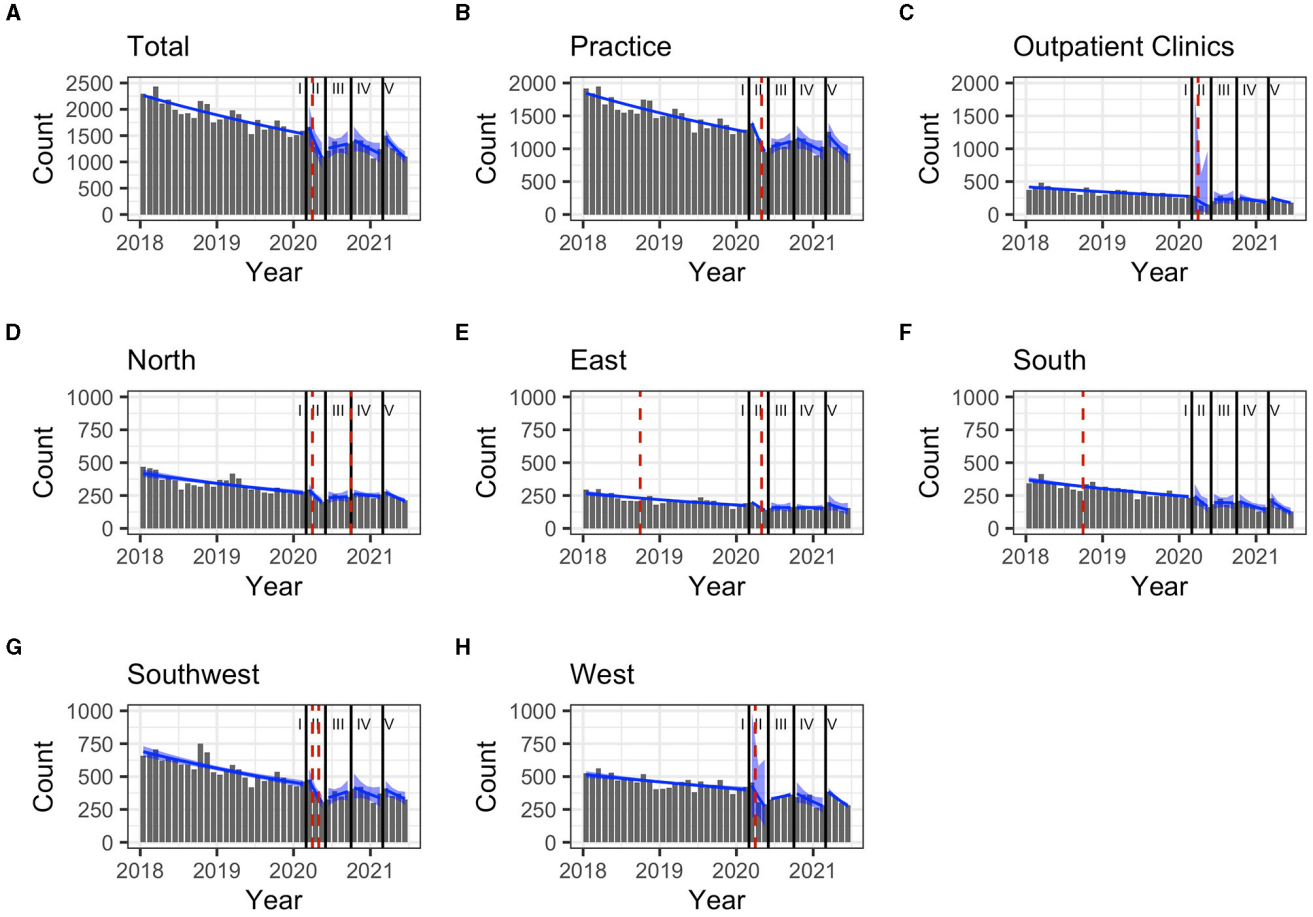
**(A–H)** Monthly prescription trends (90% confidence interval as blue band) and breakpoints (red dashed line) for Hepatitis C treatment prescriptions in Germany between January 2018 and June 2021, stratified by pandemic periods Regions: South: Bavaria; Southwest: Baden-Württemberg, Saarland, Rhineland Palatinate, Hesse; West: North Rhine, Westphalia-Lippe; North: Lower Saxony, Bremen, Hamburg, Schleswig-Holstein, Mecklenburg-Vorpommern; East: Thuringia, Saxony, Saxony-Anhalt, Brandenburg, Berlin. Pandemic phases: I: Pre-pandemic (January 2018 to February 2020), II: First pandemic wave (March 2020 to May 2020), II: Summer 2020 (June 2020 to September 2020), IV: Second wave (October 2020 to February 2021), V: Third wave (March 2021 to June 2021).

During the first pandemic wave, the total observed prescriptions stayed below predictions in total count. When stratifying for the clinical settings, prescriptions met predictions in private practices, but remained below predictions in hospital-based outpatient clinics. Stratified analyses by region resulted in prescriptions remaining below predictions in the Western and Southern region (Table 3, Figure 2). Similarly, the monthly trends of total prescriptions dropped most during the first pandemic wave compared to all other pandemic phases. In both clinical settings and in all region, the monthly trend decreased the most during this period (Table 2, Figure 3). These drops in prescriptions correlated with breakpoints that were found in March 2020 [total prescriptions data (*p* < 0.01)], in outpatient clinics (*p* < 0.01), in private practices (*p* = 0.03), in the Northern (*p* = 0.02), Western (*p* < 0.01) and Southwestern regions (*p* < 0.01) (Figure 3). Additional breakpoints were observed in April 2020, when monthly prescription trends reversed from decreasing (during the first wave) to increasing (during Summer 2020) in the Eastern (*p* = 0.053) and Southwestern region (*p* = 0.01).

During summer 2020, total prescriptions met the predictions. When stratifying for regions or for clinical settings, DAA prescriptions were also within the prediction interval (Table 3, Figure 2). During this period, the monthly trends increased for the total DAA drug prescriptions. When stratifying for clinical settings, the monthly trends increased for private practices and outpatient clinics. When stratifying for regions, the trends in the Western and Southwestern region increased, while no monthly changes were observed in the Northern, Southern and Eastern regions. Before, the monthly trends had always decreased since January 2018 (Table 2, Figure 3). Prescriptions no longer increased after the end of summer 2020 which resulted in a breakpoint (*p* = 0.02) in the Northern region in September 2020.

During the second wave, total prescriptions met predictions. When stratifying for regions, prescriptions in the North, East and Southwest met predictions (Table 3, Figure 2) but prescriptions in the Western and Southern region dropped below the prediction interval. When stratifying for clinical settings, DAA prescriptions stayed within the prediction interval, however, in outpatient clinics they fell within the lower realms of predictions. Similar to the first pandemic wave, the monthly prescriptions trends decreased more during the second wave than during the pre-pandemic phase, however to a less severe extent. The same was found when stratifying for regions: in all regions a strong negative monthly trend was observed. Equally in all clinical settings, the monthly decrease was stronger than before the pandemic (Table 2, Figure 2). However, trends fell less than during the first wave. No breakpoints were found, indicating that trends did not change abruptly.

During the third wave, the total prescriptions fell within the prediction interval (Table 3, Figure 2), despite high COVID-19 case numbers during the third wave (Supplementary material 1). When stratifying for clinical settings, DAA prescriptions met predictions. When stratifying for the region, prescriptions were within the prediction interval except for the Southern region. In the Eastern region, prescriptions even exceeded predictions. However, monthly prescription trends reached their second lowest point throughout the study period (Table 2, Figure 3). In the Northern and Southern region, monthly trends were nearly as low as during the first wave. No breakpoints were found, indicating that even though trends decreased strongly no sudden changes occurred.

## 4. Discussion

We observed a declining trend of HCV antiviral drug prescriptions from 2015 to 2021. A general decline of the drug prescriptions, even before the COVID-19 pandemic, could have affected DAA prescriptions. However, the decrease in yearly treated patients from 2019 to 2020 surpassed the decline from 2018 to 2019, indicating an additional effect of control measures to contain the COVID-19 pandemic. However overall, the number of DAA prescriptions in Germany decreased during the COVID-19 pandemic but stayed within the lower realms of predictions except for the first pandemic wave. This may be due to some specifics of the German health care system where specialist care for all patients is provided by hospital-based outpatient clinics and the private sector as a second tier.

### 4.1. Hepatitis C care between March 2020 and June 2021

A total interruption of hepatitis C care was expected and assumed in some models (17). Instead, treatment prescriptions for hepatitis C continued in Germany during all pandemic months (Figure 2). Despite a decrease in hepatitis C case notifications compared to pre-pandemic years (14, 27), HCV infections were diagnosed and notified every month throughout all pandemic periods: While hepatitis C diagnosis and treatment were impacted, they were never fully interrupted at a monthly level in Germany at any point in time during the pandemic.

Yet, our assessment confirms other observations of impaired clinical services for hepatitis C in many countries, including Germany, with the highest impact occurring during the first pandemic wave, February to May 2020 (16, 18, 28). During the first pandemic wave, prescriptions in Germany even fell below predictions (Table 3). The restrictions during the first pandemic wave in Germany also impacted on services in places where key populations, e.g., PWID, could access HCV testing: a survey among low threshold drug services showed that they were disproportionately disrupted during the first wave, and recovered only slowly (29). During the second and third pandemic wave, prescription trends differed from the pre-pandemic trend, indicating that services may still have been affected by the pandemic and related restrictions, but they were not completely shut down. This implies that patient care had not completely recovered to the pre-pandemic situation, even though physicians and patients had developed some contingency measures, e.g. video consultations replacing face-to-face visits (18). However, the monthly trends were nearly as low as in the first pandemic wave, suggesting that a longer duration of the second and third pandemic waves could have made DAA prescriptions fall below predictions.

In Germany, the COVID-19 pandemic reduced DAA prescriptions in hospital-based outpatient services more than in practices, also mainly during the first pandemic wave. It is likely that a number of different factors affected the services in outpatient clinics, leading to lower treatment prescriptions than expected and contributing to a world-wide decrease in the number of treated hepatitis C patients (28, 30): Outpatient clinics limited their service availability (18); politically imposed restrictions limited the accessibility of hospital-based outpatient care, e.g. by imposing SARS-CoV-2 PCR-tests before visits or travel restrictions; patients' fear and anxiety of contracting a SARS-CoV-2 infection in health facilities reduced their health-care seeking behavior (18). Further, the delay of elective in-patient care and limited access to in-patient care (31) possibly also reduced the service availability and access to outpatient clinics based in hospitals. In Germany, the primary care, such as private practices, played an important role in sustaining access to HCV treatment because services were less affected than in outpatient clinics.

Most other countries reported stronger reductions in hepatitis C services and DAA prescriptions than observed in Germany. A recent survey by the European Association for the Study of the Liver (EASL) (32) among clinical centers in European and non-European countries showed the impact of COVID-19 on newly initiated HCV antiviral treatments: Twenty-nine of 31 centers (94%) reported a reduction of 52% antiviral treatments in 2020 compared to 2019. In Germany, the number of initiated HCV treatments decreased only by 20% from 2019 to 2020. In the United States of America (USA), depending on the data source, the number of DAA prescriptions decreased by 22.7% (33) or 31.0% (34) in April 2020 and by 39.6% in May–July 2020 compared to the average of the same periods in 2018 and 2019 (33). During the first pandemic year (March 2020–February 2021), the number of DAA prescriptions decreased by 25.8% compared to March 2019-February 2020 (35). In Germany, prescriptions decreased by 35% in April 2020 and by 34% in May–July 2020, compared to the respective average of the years 2018 and 2019. During the first pandemic year, the number of DAA prescriptions decreased by 23%. Antiviral treatment during the pandemic declined less in Germany than in most of the countries that were represented in the EASL survey.

At a regional level, we observed that prescriptions remained below predictions in the Southern and Western region during the first and second pandemic waves. The regional 7-day COVID-19 incidence peaked higher in these two regions during the first pandemic wave (Supplementary material 2) which could have impacted the health-care seeking behavior and the HCV care availability. However, the prescriptions in the Southwestern region fell within the lower realms even though the peak of the regional 7-day COVID-19 incidence fell between the peaks observed in the Southern and Western regions. Additional factors apart from the regional 7-day COVID-19 incidence likely have affected the prescription numbers.

### 4.2. HCV elimination in Germany

Germany has committed to the international WHO 2030 elimination targets set by WHO, defined by 80% of the diagnosed to be successfully treated. Nonetheless, treatment prescriptions have steadily declined since 2015. We assume that all patients from former waiting-lists were treated, when DAAs came on the market. Even though the HCV treatment numbers before the pandemic (2014-20: *n* = 76,400) exceeded largely the number of notified newly diagnosed HCV cases (*n* = 36,514) (14), we have to assume that transmission is ongoing as new patients are diagnosed each year, and not all prevalent, longstanding infections are diagnosed. Therefore, the decline in DAA prescriptions since 2015 with an aggravation during the pandemic threatens the goal to eliminate HCV by 2030 in Germany.

According to an international modeling study (36), Germany can achieve the elimination goals if on average 9,900 are treated per year as of 2020. However, on average only 66% of the targeted patients were treated in 2020 (total treated: 6,500) (14) and only 57% in 2021 (total treated: 5,600) (37). Those 2 years with less patients treated than needed, leaves a treatment gap of 7,700 patients. To treat those patients missed in 2020 and 2021 in order to reach the treatment goals by 2029 as anticipated (36). Some additional cases might have been treated in prisons or through social welfare, private health care insurance and thus missed by our assessment. However, these were likely not enough to reach the estimated 9,900 treated cases per year necessary for HCV elimination. Other studies confirm that Germany may not reach the elimination goals by 2030: One study (38) predicts that Germany will reach HCV elimination goals by 2033, extrapolated with data from 2020. A third study (39) underlines that the declining trend of DAA prescriptions reduced the possibility that Germany reaches elimination already before the data presented here from the COVID-19 pandemic. The set-back was further aggravated by the pandemic in 2020 (39). Despite expanding the German DAA treatment indication in November 2020, which allows to prescribe DAA to all viremia patients irrespective of clinical signs of acute or chronic HCV infection (12), no trend change occurred at this point, nor did the decreasing trend flatten. It seems likely that the negative pandemic impact potentially overlaid the positive effect of extending the treatment indication. We might need more data on DAA prescription over a longer period of time to see the actual effect of the broader treatment indication. Additionally, more measures are necessary to increase treatment numbers.

We assume that the declining group of hepatitis C patients already engaged in medical care explains most of the negative trend since the peak of DAA prescriptions in 2015. Studies show that there is still a large pool of more difficult to reach infected patients, in particular among key populations (5, 40, 41), with a high proportion of long-standing chronic infections, and ongoing transmission. Possibly, the established HCV treatment services do not reach these populations effectively. In addition the pandemic caused a temporary disruption of medical services, but also of low threshold HCV prevention and care services for PWID with long standing effects (18, 29). Monthly DAA prescription trends dropped below the pre-pandemic period. Targeted interventions are needed to reduce barriers and to improve access to HCV testing and linkage to treatment for groups with a higher risk of HCV infection, e.g., PWID, people in prison, homeless people, people without health insurance.

The decrease in DAA prescriptions resulted in a delay in treating HCV infections with major implications for the patients' health and the health-care systems. A modeling study (42) estimated that the decrease in DAA prescriptions during the pandemic in 2020 increased the number of cases of liver-related deaths, hepatocellular carcinoma and decompensated cirrhosis by the factor 1.4, 1.2 and 1.5 in Spain, causing high additional costs for treatment of late-stage liver disease and cancer. It was estimated that treating the additional number of patients with a decompensated cirrhosis or cancer are associated with costs of ~4.8 million euros (42). The authors advocate for reinforcement and screening programs to reduce the excess hepatitis C morbidity and mortality related to treatment delay during the COVID-19 pandemic (42) In 2021, the German statutory health insurances included a new screening program for HCV infections for adults with the age of 35 years or older in the general health check-up which is free of charge (43). This may help to catch up the delay in diagnosing HCV infection during the pandemic to reduce the occurrence of long-term consequences of undiagnosed hepatitis C among patients engaged in medical care. Still, Germany should also implement measures to increase the diagnosis and treatment with DAA, especially for key populations.

### 4.3. Limitations

This study only represents data from patients with statutory health insurance, and patients with private insurance; those treated as refugees or asylum seekers or patients treated in prison settings were not represented. However, 88% of the German population is covered by statutory health insurance. Moreover, the aim of our study was to assess prescriptions over time and the influence of the pandemic hereof and we believe that the proportion of those treated but not covered in our data has remained constant during the study period. Further, some specialized high-selling pharmacies have refused to share their data with InsightHealth^TM^ which resulted in an estimated loss of ~5% of DAA data as of 2019. Overall, selection bias should not have impaired the validity of the findings.

The data set that we used for this analysis was anonymized and represented data of redeemed prescriptions at pharmacies. Information on the clinical setting was derived from information on unique doctor numbers, however they did not always allow to differentiate hospital-based outpatient settings from private practices. Therefore, we might have underestimated prescriptions from hospital-based outpatient clinics, and overestimated those from private practices to a small extent. The actual proportion of patients receiving an eight-week regimen of Glecaprevir + Pibrentasvir may slightly differ from our assumption, resulting in a few more or less HCV patients treated per year, which we assumed to be negligible. The data set lacks information on patient data, such as age, gender, reinfection with HCV, number of previous treatment courses with DAA, migration status or HIV status, thus impeding the assessment of trends among different age groups or the analysis of how the COVID-19 pandemic affected different sub-populations.

We restricted the pre-pandemic period to January 2018 to February 2020. This limited the number of data point, especially because the prescription data was only available at a monthly level. Therefore, we refrained from doing a time-series analysis to avoid overfitting of the pre-pandemic data. We did not include more months in the pre-pandemic period as we saw unsystematic changes in prescriptions prior to January 2018, likely due to the approval of new drugs or drug combinations, which would have impaired the validity of our regression models.

### 4.4. Recommendations

We need further studies to understand the underlying reasons for the regional differences in DAA prescriptions. Comparing the time between HCV diagnosis and treatment initiation before and during the COVID-19 pandemic can shed further light on how the pandemic impacted the continuity-of-care cascade for HCV in Germany, in particular for key populations. Considering the HCV elimination goals, it is important to understand if missed patients from the first pandemic wave were treated later or if they were lost to follow-up. Furthermore, we need to learn from service providers and patients what interventions could increase the number of treated patients.

Hepatitis services need to reach more patients with hepatitis C to minimize the impacts of the pandemic on the reduced number of treated patients. The declining pre-pandemic trend suggests that easy-to-reach patients have been treated. The pandemic disproportionately reduced HCV testing and care points, like low threshold drug services, for key populations, who are simultaneously at high-risk for HCV infections, such as PWID, homeless people or people in prisons (29). Therefore, targeted interventions are necessary to improve access to testing and treatment for key populations to achieve the HCV elimination goals by 2030. This includes improving the collaboration between harm reduction services, opioid substitution facilities and infectious diseases services. Based on the current data, the number of DAA prescriptions need to increase if Germany is to reach the HCV elimination goals. Furthermore, the health-care system should implement screening services to detect patients with HCV-related complications early to minimize the additional treatment costs that the treatment delay caused during the COVID-19 pandemic.

## 5. Conclusion

Overall, the total number of DAA prescriptions was within the lower realms of predicted counts. However, during the first pandemic wave the number of prescriptions was lower than predicted and monthly trends plummeted. Monthly trends fell also below the pre-pandemic trend during the second and third wave. Practices of the primary care sector managed better to uphold services than hospital-based outpatient clinics. The decline in the number of DAA prescriptions during the pandemic was a global phenomenon. DAA treatment declined less in Germany than compared to most countries represented in the EASL survey. However, the additional decrease in the number of DAA prescriptions due to the pandemic could reduce the possibility of Germany reaching the HCV elimination goal with 90% of infected diagnosed, and 80% of diagnosed eligible patients treated by 2030. To achieve these targets, more HCV-infected patients, including key populations, need to be reached, diagnosed and treated. The delay in treating hepatitis C during the pandemic will cause additional patients with HCV-related complications. Screening programs should aim to diagnose these patients early and link them to specific care to reduce additional costs.

## Data availability statement

The data analyzed in this study is subject to the following licenses/restrictions: The data analyzed in this study was obtained from InsightHealth^TM^, the following restrictions apply: purchasers commercial license restrictions. Requests to access these datasets should be directed to CK, KollanC@rki.de.

## Author contributions

Study conception, protocol development, and manuscript preparation: EM, SD, CK, DS, and RZ. Data collection: CK. Data analysis: EM and CK. All authors contributed to the article and approved the submitted version.
