# Supplementary material for: Additional challenges in reaching hepatitis C elimination goals in Germany due to the COVID-19 pandemic - descriptive analysis of drug prescription data from January 2018 to June 2021
Source: Front Public Health. 2023 May 30;11:1149694. doi: 10.3389/fpubh.2023.1149694 (PMC10267983; doi:10.3389/fpubh.2023.1149694)
Supplement: Supplementary file 1 [file Data_Sheet_1.docx]

Supplementary Material

Reaching hepatitis C elimination goals in Germany by 2030 is challenged by a decrease in treatment numbers, exacerbated by the first COVID-19 pandemic wave- Descriptive analysis of drug prescription data from January 2018 to June 2021

**Emily Meyer, Sandra Dudareva, Christian Kollan, Stefan Mauss, Heiner Wedemeyer, Daniel Schmidt, Ruth Zimmermann^*^**

*** Correspondence:** Corresponding Author: Ruth Zimmermann, zimmermannr@rki.de

## Supplementary Figures

**Supplementary Figure 1.** COVID-19 cases (fulfilling the reference definition) and pandemic phases in Germany, January 2020 to June 2021


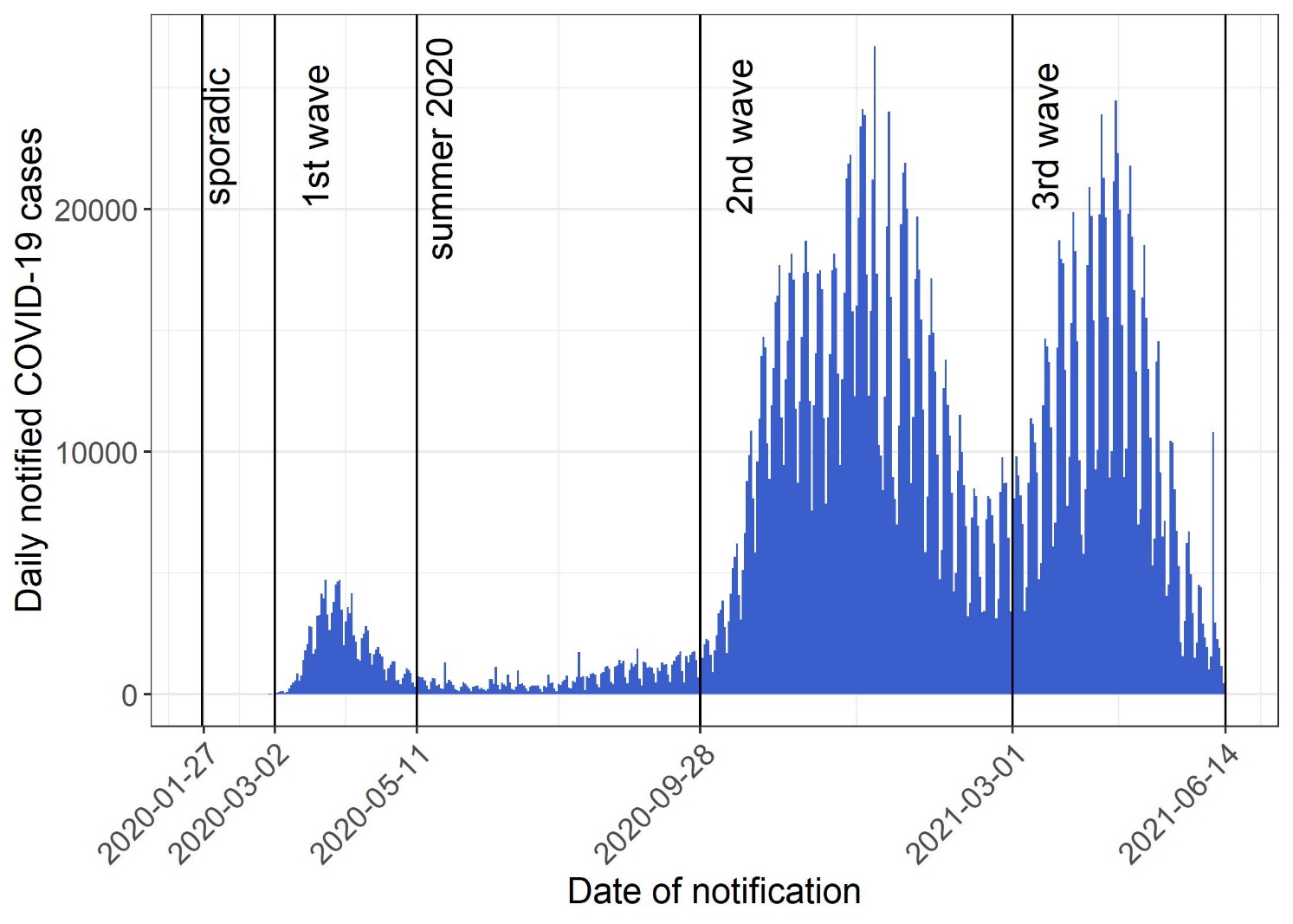


**Supplementary Figure 2.** 7-day COVID-19 incidence per region in Germany, March 2020 to June 2021

South: Bavaria, Southwest: Baden-Württemberg, Saarland, Rhineland Palatinate, Hesse, West: North Rhine, Westphalia-Lippe, North: Lower Saxony, Bremen, Hamburg, Schleswig-Holstein, Mecklenburg-Vorpommern, East: Thuringia, Saxony, Saxony-Anhalt, Brandenburg, Berlin

Panel A


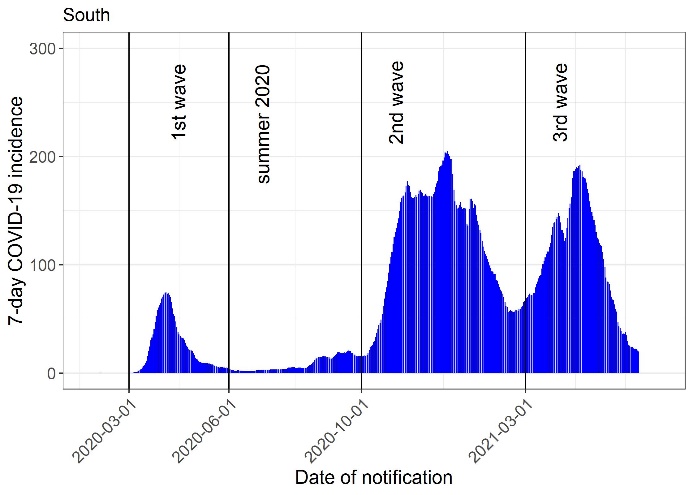


**Panel B**


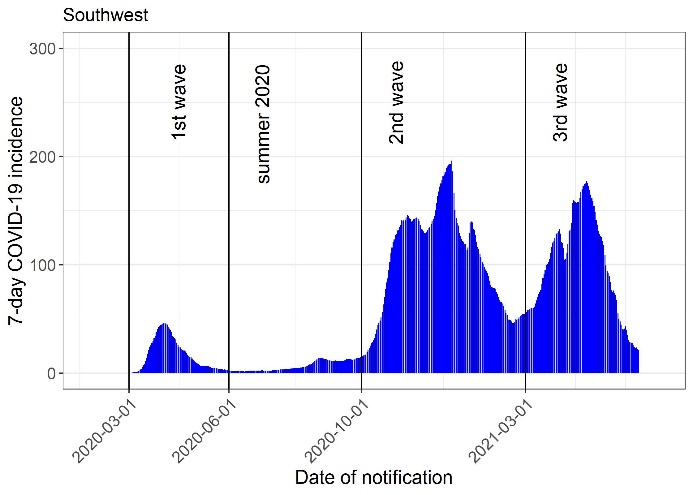


**Panel C**


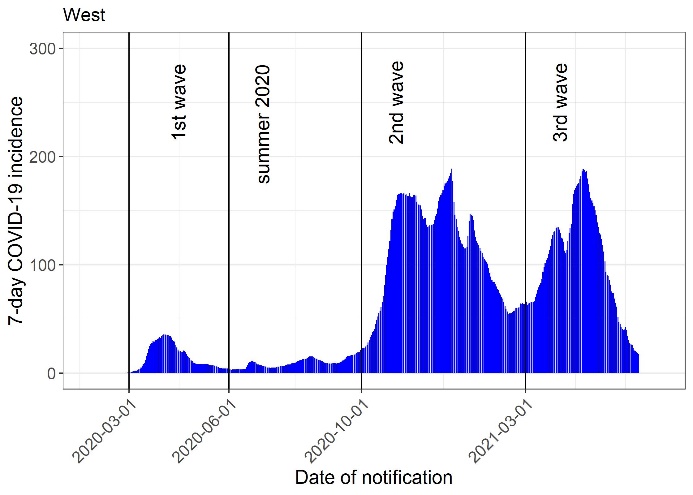


**Panel D**


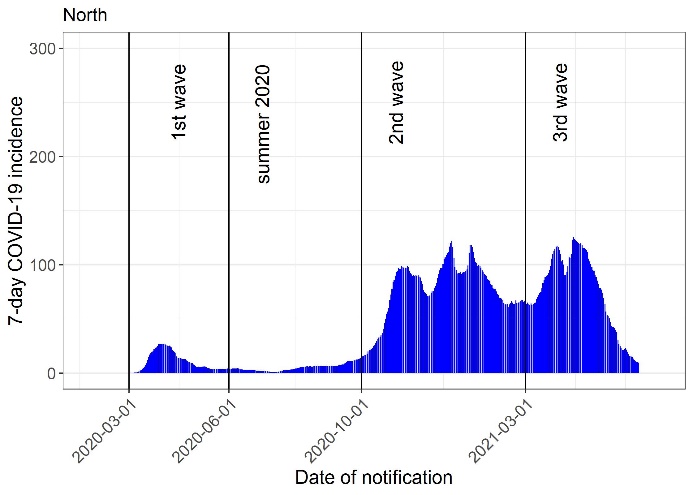


**Panel E**


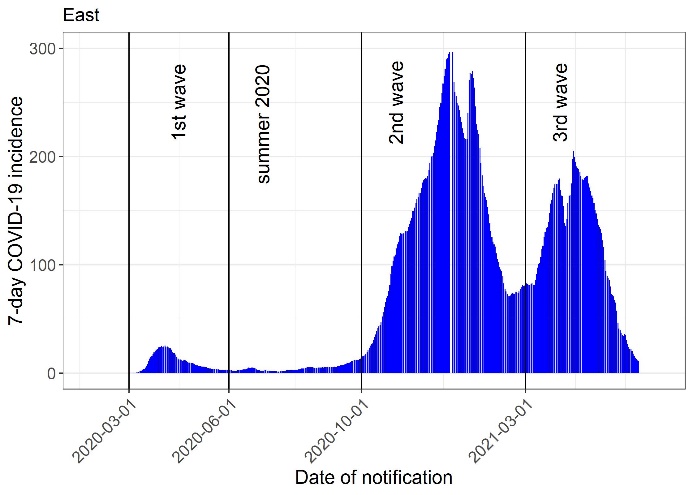


**Supplementary Table 1.** Prescription of DAA substances and combinations in Germany between January 2018 and June 2021, total number and row percentages

|  |  |  |  |  |  |  |  |  |  |  |
| --- | --- | --- | --- | --- | --- | --- | --- | --- | --- | --- |
| Year | Month | Glecaprevir+ Pibrentasvir | Velpatasvir + Sofosbuvir | Grazoprevir + Elbasvir | Ledipasvir+  Sofosbuvir | Voxilaprevir+ Velpatasvir+ Sofosbuvir | Sofosbuvir | Paritaprevir+ Ombitasvir+ Ritonavir | Dasabuvir | Daclatasvir |
| 2018 | 1 | 1113 (48.56%) | 400 (17.45%) | 396 (17.28%) | 215 (9.38%) | 149 (6.5%) | 6 (0.26%) | 7 (0.31%) | 6 (0.26%) |  |
| 2018 | 2 | 1231 (54.44%) | 379 (16.76%) | 366 (16.19%) | 160 (7.08%) | 110 (4.87%) | 7 (0.31%) | 4 (0.18%) | 3 (0.13%) | 1 (0.04%) |
| 2018 | 3 | 1208 (49.63%) | 468 (19.23%) | 471 (19.35%) | 141 (5.79%) | 130 (5.34%) | 7 (0.29%) | 5 (0.21%) | 3 (0.12%) | 1 (0.04%) |
| 2018 | 4 | 1054 (50.02%) | 387 (18.37%) | 435 (20.65%) | 118 (5.6%) | 97 (4.6%) | 8 (0.38%) | 4 (0.19%) | 2 (0.09%) | 2 (0.09%) |
| 2018 | 5 | 994 (45.55%) | 478 (21.91%) | 486 (22.27%) | 116 (5.32%) | 94 (4.31%) | 9 (0.41%) | 1 (0.05%) | 1 (0.05%) | 3 (0.14%) |
| 2018 | 6 | 842 (42.21%) | 471 (23.61%) | 475 (23.81%) | 102 (5.11%) | 83 (4.16%) | 15 (0.75%) | 1 (0.05%) | 1 (0.05%) | 5 (0.25%) |
| 2018 | 7 | 815 (42.67%) | 478 (25.03%) | 458 (23.98%) | 78 (4.08%) | 71 (3.72%) | 5 (0.26%) | 2 (0.1%) | 2 (0.1%) | 1 (0.05%) |
| 2018 | 8 | 815 (42.4%) | 511 (26.59%) | 453 (23.57%) | 76 (3.95%) | 60 (3.12%) | 6 (0.31%) |  |  | 1 (0.05%) |
| 2018 | 9 | 763 (41.6%) | 537 (29.28%) | 406 (22.14%) | 70 (3.82%) | 52 (2.84%) | 5 (0.27%) |  |  | 1 (0.05%) |
| 2018 | 10 | 922 (42.78%) | 609 (28.26%) | 504 (23.39%) | 68 (3.16%) | 49 (2.27%) | 3 (0.14%) |  |  |  |
| 2018 | 11 | 885 (42.12%) | 600 (28.56%) | 511 (24.32%) | 67 (3.19%) | 35 (1.67%) | 3 (0.14%) |  |  |  |
| 2018 | 12 | 745 (42.52%) | 510 (29.11%) | 396 (22.6%) | 63 (3.6%) | 34 (1.94%) | 1 (0.06%) |  | 1 (0.06%) | 2 (0.11%) |
| 2019 | 1 | 737 (40.88%) | 537 (29.78%) | 425 (23.57%) | 56 (3.11%) | 46 (2.55%) |  |  | 2 (0.11%) |  |
| 2019 | 2 | 777 (42.09%) | 550 (29.79%) | 420 (22.75%) | 46 (2.49%) | 50 (2.71%) | 1 (0.05%) | 1 (0.05%) | 1 (0.05%) |  |
| 2019 | 3 | 751 (37.87%) | 635 (32.02%) | 492 (24.81%) | 34 (1.71%) | 70 (3.53%) | 1 (0.05%) |  |  |  |
| 2019 | 4 | 707 (37.13%) | 624 (32.77%) | 467 (24.53%) | 43 (2.26%) | 62 (3.26%) | 1 (0.05%) |  |  |  |
| 2019 | 5 | 688 (38.48%) | 586 (32.77%) | 424 (23.71%) | 33 (1.85%) | 57 (3.19%) |  |  |  |  |
| 2019 | 6 | 569 (37.12%) | 538 (35.09%) | 352 (22.96%) | 28 (1.83%) | 45 (2.94%) | 1 (0.07%) |  |  |  |
| 2019 | 7 | 687 (38.29%) | 597 (33.28%) | 423 (23.58%) | 41 (2.29%) | 44 (2.45%) | 2 (0.11%) |  |  |  |
| 2019 | 8 | 676 (41.86%) | 517 (32.01%) | 351 (21.73%) | 24 (1.49%) | 45 (2.79%) | 2 (0.12%) |  |  |  |
| 2019 | 9 | 722 (43.63%) | 517 (31.24%) | 346 (20.91%) | 33 (1.99%) | 35 (2.11%) | 2 (0.12%) |  |  |  |
| 2019 | 10 | 787 (43.99%) | 570 (31.86%) | 371 (20.74%) | 22 (1.23%) | 36 (2.01%) | 3 (0.17%) |  |  |  |
| 2019 | 11 | 741 (44.13%) | 520 (30.97%) | 341 (20.31%) | 33 (1.97%) | 40 (2.38%) | 4 (0.24%) |  |  |  |
| 2019 | 12 | 588 (39.86%) | 537 (36.41%) | 283 (19.19%) | 24 (1.63%) | 41 (2.78%) | 2 (0.14%) |  |  |  |
| 2020 | 1 | 659 (43.61%) | 498 (32.96%) | 297 (19.66%) | 24 (1.59%) | 30 (1.99%) | 3 (0.2%) |  |  |  |
| 2020 | 2 | 724 (45.51%) | 509 (31.99%) | 298 (18.73%) | 29 (1.82%) | 29 (1.82%) | 2 (0.13%) |  |  |  |
| 2020 | 3 | 692 (41.49%) | 569 (34.11%) | 350 (20.98%) | 27 (1.62%) | 27 (1.62%) | 3 (0.18%) |  |  |  |
| 2020 | 4 | 549 (42.46%) | 432 (33.41%) | 268 (20.73%) | 15 (1.16%) | 23 (1.78%) | 6 (0.46%) |  |  |  |
| 2020 | 5 | 499 (45.2%) | 333 (30.16%) | 214 (19.38%) | 29 (2.63%) | 25 (2.26%) | 4 (0.36%) |  |  |  |
| 2020 | 6 | 518 (42.53%) | 402 (33%) | 235 (19.29%) | 27 (2.22%) | 32 (2.63%) | 4 (0.33%) |  |  |  |
| 2020 | 7 | 608 (43.74%) | 456 (32.81%) | 265 (19.06%) | 31 (2.23%) | 27 (1.94%) | 3 (0.22%) |  |  |  |
| 2020 | 8 | 531 (42.38%) | 433 (34.56%) | 238 (18.99%) | 20 (1.6%) | 26 (2.08%) | 5 (0.4%) |  |  |  |
| 2020 | 9 | 569 (42.05%) | 481 (35.55%) | 258 (19.07%) | 16 (1.18%) | 27 (2%) | 2 (0.15%) |  |  |  |
| 2020 | 10 | 564 (40.52%) | 512 (36.78%) | 251 (18.03%) | 21 (1.51%) | 38 (2.73%) | 6 (0.43%) |  |  |  |
| 2020 | 11 | 574 (40.74%) | 518 (36.76%) | 256 (18.17%) | 19 (1.35%) | 37 (2.63%) | 5 (0.35%) |  |  |  |
| 2020 | 12 | 561 (42.69%) | 474 (36.07%) | 218 (16.59%) | 23 (1.75%) | 36 (2.74%) | 2 (0.15%) |  |  |  |
| 2021 | 1 | 444 (41.5%) | 416 (38.88%) | 172 (16.07%) | 18 (1.68%) | 19 (1.78%) | 1 (0.09%) |  |  |  |
| 2021 | 2 | 532 (42.87%) | 479 (38.6%) | 189 (15.23%) | 25 (2.01%) | 16 (1.29%) |  |  |  |  |
| 2021 | 3 | 630 (41.89%) | 602 (40.03%) | 220 (14.63%) | 28 (1.86%) | 24 (1.6%) |  |  |  |  |
| 2021 | 4 | 527 (41.73%) | 512 (40.54%) | 171 (13.54%) | 31 (2.45%) | 20 (1.58%) | 2 (0.16%) |  |  |  |
| 2021 | 5 | 519 (44.36%) | 456 (38.97%) | 142 (12.14%) | 18 (1.54%) | 32 (2.74%) | 3 (0.26%) |  |  |  |
| 2021 | 6 | 443 (39.98%) | 457 (41.25%) | 168 (15.16%) | 10 (0.9%) | 28 (2.53%) | 2 (0.18%) |  |  |  |
